# NH_3_/NH_4_^+^ allosterically activates SLC4A11 by causing an acidic shift in the intracellular pK that governs H^+^(OH^−^) conductance

**DOI:** 10.3389/fphys.2024.1440720

**Published:** 2024-08-14

**Authors:** Richard A. Pasternack, Bianca N. Quade, Aniko Marshall, Mark D. Parker

**Affiliations:** ^1^ Department of Physiology and Biophysics, The State University of New York: The University at Buffalo, Buffalo, NY, United States; ^2^ Department of Ophthalmology, The State University of New York: The University at Buffalo, Buffalo, NY, United States

**Keywords:** acid-base, Btr1, NaBC1, cornea, proton

## Abstract

SLC4A11 is the most abundant membrane transport protein in corneal endothelial cells. Its functional presence is necessary to support the endothelial fluid pump that draws fluid from the corneal stroma, preventing corneal edema. Several molecular actions have been proposed for SLC4A11 including H_2_O transport and cell adhesion. One of the most reproduced actions that SLC4A11 mediates is a H^+^ (or OH^−^) conductance that is enhanced in the presence of NH_4_Cl. The mechanism by which this occurs is controversial with some providing evidence in favor of NH_3_-H^+^ cotransport and others providing evidence for uncoupled H^+^ transport that is indirectly stimulated by the effects of NH_4_Cl upon intracellular pH and membrane potential. In the present study we provide new evidence and revisit previous studies, to support a model in which NH_4_Cl causes direct allosteric activation of SLC4A11 by means of an acidic shift in the intracellular pK (pK_i_) that governs the relationship between intracellular pH (pH_i_) and SLC4A11 H^+^-conductance. These findings have important implications for the assignment of a physiological role for SLC4A11.

## 1 Introduction

The members of the SLC4 family of solute carrier proteins are mainly Na^+^-independent and Na^+^-dependent Cl^−^/HCO_3_^−^ exchangers or Na^+^-coupled HCO_3_^−^ (or CO_3_^=^) transporters (Parker and Boron, 2013; Lee et al., 2022). SLC4A11 (originally named “BTR1”: Bicarbonate Transporter Related Protein 1) was the last member of the SLC4 family to be cloned (Parker et al., 2001) and is the only member of the family that does not transport HCO_3_^−^/CO_3_^=^ (Jalimarada et al., 2013; Ogando et al., 2013; Loganathan et al., 2016). Instead, SLC4A11 influences intracellular pH (pH_i_) by conducting H^+^, or its thermodynamic equivalent OH^−^ (Kao et al., 2015; Myers et al., 2016). For convenience hereafter, and in the absence of any definitive data in favor of one substrate over the other, we will assume that H^+^ are the transported species. SLC4A11 is expressed at low levels in many tissues, but appears to have most functional impact in the cornea and inner ear as evidenced by the corneal dystrophies and progressive hearing loss that are caused by SLC4A11 mutations in both humans and mice (Vithana et al., 2006; 2008; Desir et al., 2007; Lopez et al., 2009; Gröger et al., 2010; Han et al., 2013).

Most studies of SLC4A11 have focused on its role in the cornea. Here, SLC4A11 is expressed in the basolateral membrane of the corneal endothelial cells that line the posterior (aqueous-humor facing side) of the cornea (Vilas et al., 2013). These cells are responsible for pumping fluid from the corneal stroma to prevent it from swelling and losing its ability to optimally transmit and refract light (Hodson, 1977). SLC4A11 is clearly valuable for endothelial pumping because autosomal recessive inheritance of SLC4A11 mutations cause congenital hereditary endothelial dystrophy (CHED), a non-progressive corneal thickening and opacification (Vithana et al., 2006). Furthermore, an apparently discrete set of SLC4A11 mutations result in autosomal dominant inheritance of a late-onset form of Fuchs endothelial corneal dystrophy (FECD4), which typically manifests from the 4th decade of life, preceded by the appearance of excrescences from the Descemet’s membrane that underlie the endothelial cells, which are known as guttae (Weiss et al., 2024). However, the mechanism by which SLC4A11 loss compromises the endothelial pump remains elusive because there is little consensus about SLC4A11’s molecular action.

The earliest proposal for SLC4A11 action was that it, like plantal Slc4-like proteins, performed borate transport: specifically electrogenic 2Na^+^-B(OH)_4_^–^ cotransport (Frommer and von Wirén, 2002; Park et al., 2004). This hypothesis fell out of favor due to an inability of others to find evidence for this mode of action with mammalian SLC4A11 (Jalimarada et al., 2013; Ogando et al., 2013; Vilas et al., 2013; Kao et al., 2015; Loganathan et al., 2016). That original characterization study also attributed an EIPA-insensitive Na^+^/H^+^ exchanger-like activity to SLC4A11, but neither the EIPA-sensitivity nor the Na^+^-dependence of this H^+^ transport activity has proven to be universally repeatable, leaving open the possibility that such results were influenced by endogenous activities (Park et al., 2004; Ogando et al., 2013; Kao et al., 2015; 2016; Zhang et al., 2015; Myers et al., 2016). Other reported SLC4A11 transport modes include a H_2_O permeability that is disturbed by disease-causing mutations (Vilas et al., 2013), a role in extracellular matrix adhesion (Malhotra et al., 2020), and a conditional presence in mitochondria (Ogando et al., 2019). Any of these, alone or in combination, remain a feasible explanation for loss of endothelial pump function with SLC4A11 mutation. However, at present, none of these features has been investigated by more than one group.

In this study, we focus on the robust and repeatable Na^+^-independent H^+^ transport action of SLC4A11, an action that is stimulated by increases in pH_i_, extracellular pH (pH_e_), and NH_4_Cl (Zhang et al., 2015; Myers et al., 2016; Kao et al., 2020; Quade et al., 2020) and which is also disrupted by disease-causing mutations (Kao et al., 2015; Quade et al., 2022). However, there remains a controversy over whether the NH_4_Cl-stimulated action of SLC4A11 represents SLC4A11-mediated NH_3_-H^+^ cotransport (Zhang et al., 2015; Kao et al., 2020) or an indirect stimulation of SLC4A11-mediated H^+^ conductance caused by cellular depolarization and sub-membranous alkalinization of pH_i_ due to NH_3_ movement across the lipid bilayer (Myers et al., 2016). A recent attempt to distinguish NH_3_-H^+^ cotransport from NH_4_^+^ or H^+^ transport using the Goldman-Hodgkin-Katz approach led to the conclusion that SLC4A11 operates in competing modes of NH_3_-H^+^ cotransport or unaccompanied H^+^ transport, with the inference that the presence of NH_3_/NH_4_^+^ inhibits unaccompanied H^+^ conduction (Kao et al., 2020). These are important distinctions as they inform the predicted direction of transport, the interpretation of SLC4A11 structure, and the ultimately the physiological role of SLC4A11. In our previous work, we demonstrated that SLC4A11-mediated H^+^ transport is governed by an intracellular pK (pK_i_), the value of which can be modulated by changes in pH_e_ and by disease-causing mutations (Quade et al., 2020; Quade et al., 2022). At pH_e_ = 7.50, pK_i_ is too alkaline to be determined, but can be no more acidic than 7.6 (Quade et al., 2020). However, when we raise pH_e_, pK_i_ is shifted into measurable range. For example, pK_i_ for human SLC4A11 ∼ 7.04 when pH_e_ = 8.50 (Quade et al., 2022). This acidic-shift in pK_i_ manifests as a rise in SLC4A11 current. We note that no study of SLC4A11 activity has directly measured transmembrane NH_3_/NH_4_^+^ movement, relying instead on proxies such as pH and voltage changes. With that as context, here we revisit the phenomenon of NH_4_Cl-stimulation of SLC4A11-mediated H^+^ currents to determine whether it could be explained by a direct allosteric effect of NH_3_/NH_4_^+^ upon SLC4A11 pK_i_.

## 2 Results

### 2.1 Determining pK_i_ for SLC4A11 at pH_e_ = 8.50

**FIGURE 1:**
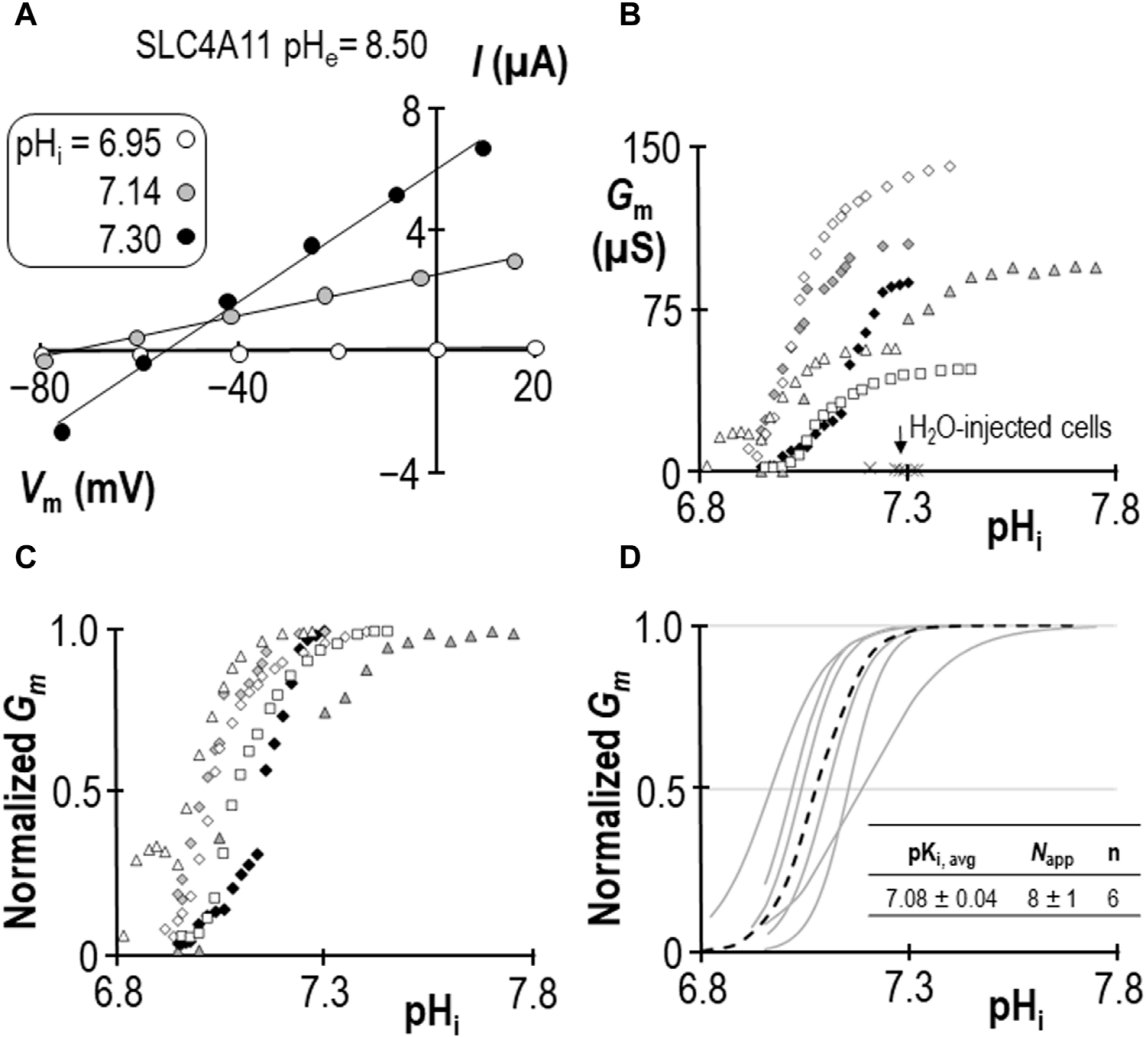
SLC4A11 behavior in the absence of NH_4_Cl (extracellular pH = 8.50) **(A)** A representative selection of current-voltage (I-V) relationships gathered from a single SLC4A11-expressing oocyte as intracellular pH (pH_i_) rises. **(B)** The relationship between pH_i_ and slope conductance (*G*_m_) from the full-set of I-V relationships gathered from the SLC4A11-expressing cell shown in panel (A) (black diamonds) and from five other SLC4A11-expressing cells (each represented by its own symbol). The pH_i_ versus *G*_m_ relationship at resting pH_i_ for five H_2_O-injected oocytes is represented by crosses. **(C)** SLC4A11 data from panel (B), normalized to its respective maximum *G*_m_ (*G*_m,max_). **(D)** Best-fit lines for each cell to the Hill equation are shown in gray. The dashed black line represents the Hill equation generated using the average pK_i_ and apparent Hill coefficients (*N*_app_) of the n = 6 replicates, as shown in the inset table.

In a previous study we determined that pK_i_ for human SLC4A11 at pH_e_ = 8.50 is 7.04 ± 0.01 (Quade et al., 2022). For this study we generated a contemporary set of control data to confirm the pK_i_ of human SLC4A11 at pH_e_ = 8.50 in new experimental hands. As we have previously reported, SLC4A11-expressing oocytes slowly alkalinize upon exposure to pH_e_ = 8.50 solution, and the rate of alkalinization can be enhanced by clamping the membrane potential (*V*_m_) at a value more positive than the predicted reversal potential for H^+^ (*E*_H_). An example of this phenomenon can see seen in the first figure of Quade et al. (2022). As pH_i_ rises, we gather a series of I-V plots such that each I-V plot can be assigned to a value of pH_i_. Figure 1A shows a selection of these plots gathered from a single SLC4A11-expressing oocyte as pH_i_ is caused to rise under voltage-clamp. Note that the slope of the I-V relationship (i.e., membrane conductance, *G*_m_) rises as pH_i_ increases. The relationship between pH_i_ and *G*_m_ is shown for six SLC4A11-expressing cells in Figure 1B, in which the trace marked by black diamonds is the full data set from the cell shown in Figure 1A. The average *G*_m.max_ was 90 ± 14 µS. Also shown in Figure 1B are resting pH_i_/*G*_m_ relationships from six H_2_O-injected cells (crosses) for which the average *G*_m_ was 2 ± 1 µS. In order to extract pK_i_ values from the SLC4A11 data, we first normalize *G*_m_ data from each cell to its own *G*_m,max_ (Figure 1C) and fit those data to the Hill equation, generating the best-fit relationships described by a pK_i_ and an apparent Hill coefficient (*N*_app_) shown as gray lines in Figure 1D. The average of these relationships is represented as a black dotted-line in Figure 1. We calculate an average pK_i_ of 7.08 ± 0.04 for human SLC4A11 at pH_e_ = 8.50, which is not different from the range that we had previously determined in Quade et al. (2022) (*p* = 0.32, two-tailed, unpaired t-test).

### 2.2 Determining pK_i_ for SLC4A11 at pH_e_ = 8.50 in the presence of 1 mM NH_4_Cl

**FIGURE 2:**
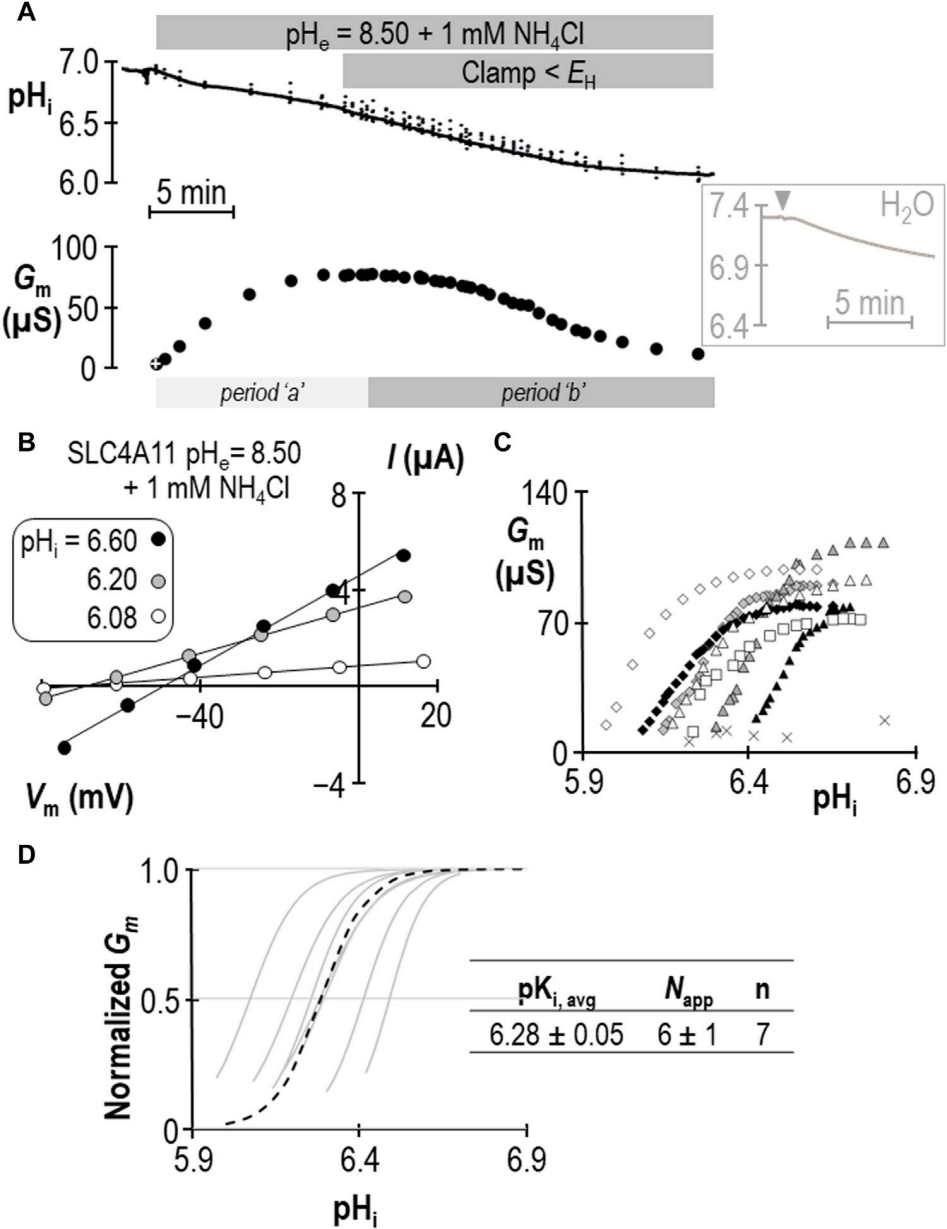
SLC4A11 behavior in the presence of 1 mM NH_4_Cl (extracellular pH = 8.50). **(A)** A representative example of the pH_i_ (top) and *G*_m_ (bottom) response of an SLC4A11-expressing oocyte to the addition of 1 mM NH_4_Cl. The first *G*_m_ point in the data series (white cross) was gathered prior to the presence of NH_4_Cl. The inset shows a representative pH_i_ response to the addition of 1 mM NH_4_Cl (point of solution change indicated by gray triangle) of a H_2_O-injected cell at pH_e_ = 8.50. **(B)** A representative selection of I-V relationships gathered from a single SLC4A11-expressing oocyte as pH_i_ falls during period “b”. **(C)** The relationship between pH_i_ and *G*_m_ from the full-set of I-V relationships gathered from the SLC4A11-expressing cell shown in panel (B) (black diamonds) and from six other SLC4A11-expressing cells (each represented by its own symbol). The pH_i_ versus *G*_m_ relationship for six H_2_O-injected oocytes after acidification by HCl injection is represented by crosses. **(D)** Best-fit lines for each cell to the Hill equation are shown in gray. The dashed black line represents the Hill equation generated using the average pK_i_ and *N*_app_ of the n = 7 replicates, as shown in the inset table.

The response of SLC4A11-expressing oocytes in pH_e_ = 8.50 solution was altered in two ways in the presence of 1 mM NH_4_Cl. First, as shown in the example in Figure 2A, the cell acidified rather than alkalinized (initial dpH_i_/dt = 5.1 ± 0.9 × 10^−4^ pH units/s, n = 6). This was also a feature of H_2_O-injected cells (initial dpH_i_/dt = 5.5 ± 0.8 × 10^−4^ pH units/s, n = 6, Figure 2A inset). Second, unique to SLC4A11-expressing cells, *G*_m_ unexpectedly rose to a plateau (over period “a”) and subsequently declined to a value close to its starting value (over period “b”) when pH_i_ was further acidified by clamping *V*_m_ at a value more negative than *E*_H_. Let us first focus our attention on period “b,” during which acidification causes a familiar decline. Figure 2B shows a selection of responses from the cell represented in Figure 2A. The pH_i_ versus *G*_m_ relationships gathered from seven SLC4A11-expressing cells during period “b” are plotted in Figure 2C. The average *G*_*m*.max_ was 89 ± 5 µS, which is not different from that for the group of cells assayed in the absence of NH_4_Cl (*p* = 0.94, two-tailed unpaired t-test). Also shown in Figure 2C are resting pH_i_/*G*_m_ relationships from six control cells (originally injected with H_2_O in place of SLC4A11 cRNA) that were acidified prior to assay by HCl injection (crosses). The average *G*_m_ of these cells was 11 ± 2 µS. Best-fit normalized *G*_m_ versus pH_i_ data for SLC4A11-expressing cells is shown in Figure 2D. We calculate that the average pK_i_ for SLC4A11-expressing cells at pH_e_ = 8.50 in the presence of 1 mM NH_4_Cl is 6.28 ± 0.05, which is significantly more acidic than the pK_i_ range determined in the absence of NH_4_Cl (*p* < 0.001, one-tailed, unpaired t-test). On the other hand, there was no significant difference in the value of *N*_app_ compared to its value in the absence of NH_4_Cl (*P* = 0.17, two-tailed unpaired t-test).

**FIGURE 3:**
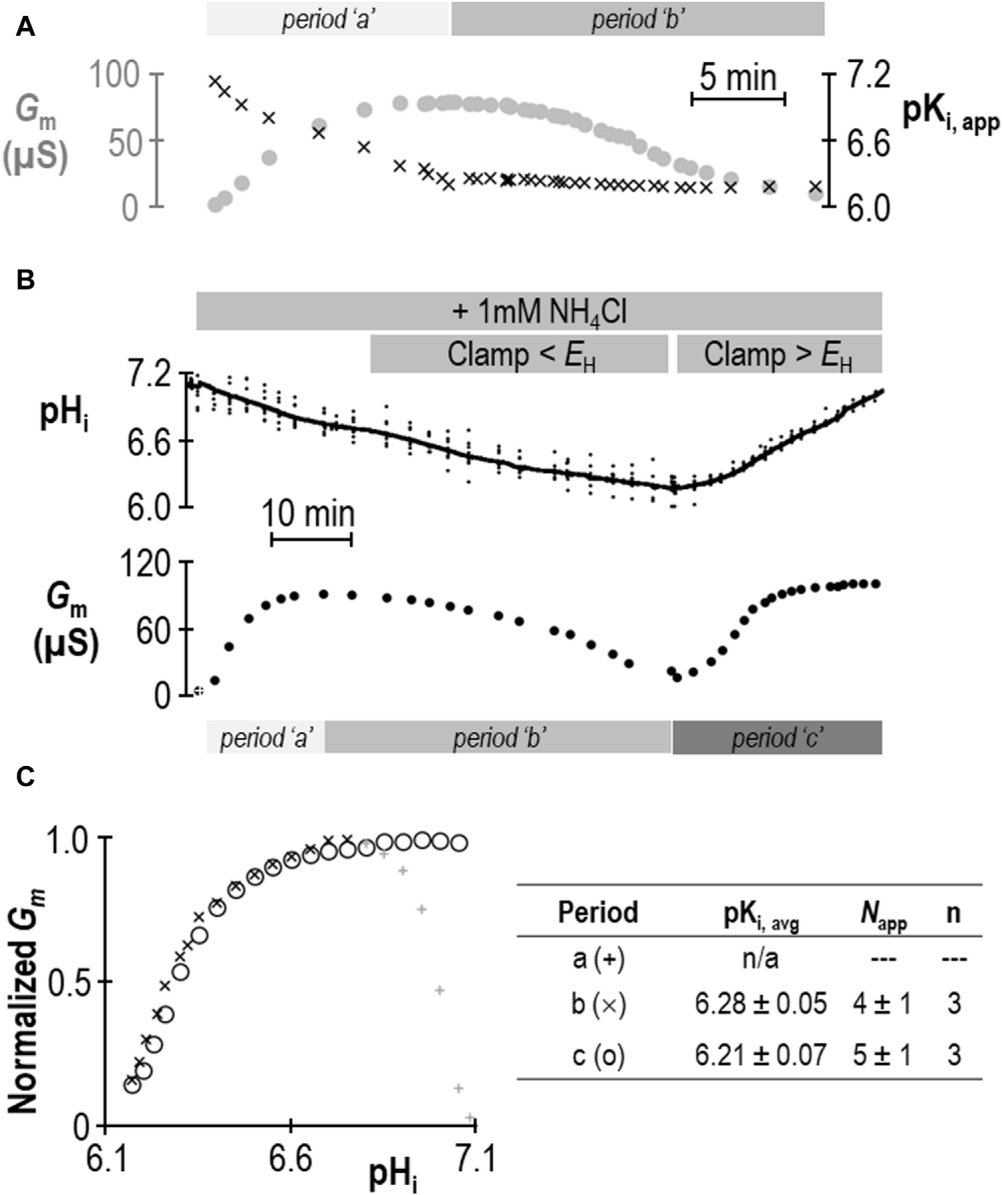
Validation of change in SLC4A11 pK_i_ upon addition of NH_4_Cl. **(A)** *G*_m_ data from Figure 2A replotted with the apparent pK_i_ (pK_i,app_) calculated from the pH_i_ at each value of *G*_m_. **(B)** A representative experiment similar to that shown in Figure 2A, extended into a third experimental period in which *G*_m_ is monitored during a return to starting pH_i_. **(C)** *G*_m_ values from panel (B) plotted for each of the three experimental periods, normalized to *G*_m,max_ for each period. The inset table shows pK_i_ and *N*_app_ for periods “b” and “c” calculated from best-fit data to the Hill equation for three such experiments.

Does the action of SLC4A11 during period “a” represent a second pK_i_ that reports acid-activation? We hypothesized that period “a” represented the time during which pK_i_ was transitioning between its ± NH_4_Cl values. If we assume that neither *G*_m,max_ nor *N*_app_ change in a single SLC4A11-expressing oocyte during the course of an experiment such as that in Figure 2A, we can solve the Hill equation to generate an apparent pK_i_ (pK_i,app_) for each point in the experiment at which we have paired values of *G*_m_ and pH_i_. The results of this approach are shown in Figure 3A and support the hypothesis that period “a” is dynamic time of pK_i_ adjustment, while period “b” represents a time over which SLC4A11 has assumed a new and relatively stable pK_i_. In three of our seven experiments, we extended the protocol to examine a period “c” (example shown in Figure 3B) during which we could examine the behavior of SLC4A11 in the pH-range of period “a”, but after SLC4A11 has assumed its + NH_4_Cl pK_i_. As shown in Figure 3C, SLC4A11 exhibits a similar pK_i_ during period “b” versus period “c” pK_i_ (*P* = 0.19: two-tailed, paired t-test) and does not revisit the apparent acid-activated behavior exhibited during period “a.” In summary for this section, we find that the presence of 1 mM NH_4_Cl results in a significant acidic shift in the value of pK_i_ for human SLC4A11 at pH_e_ = 8.50.

### 2.3 Determining the ion-selectivity of SLC4A11 in the presence of 1 mM NH_4_Cl

**FIGURE 4:**
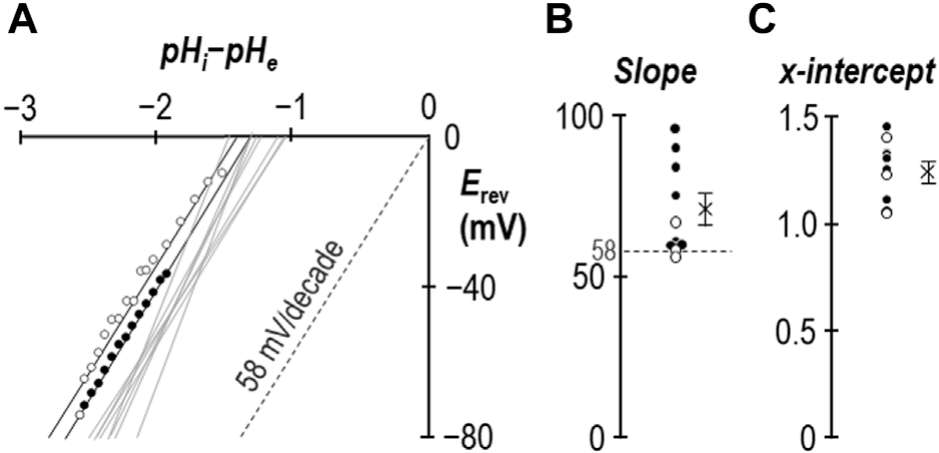
The ion-selectivity of SLC4A11 in the presence of NH_4_Cl. **(A)** The relationship between *V*_m_ and the transmembrane pH gradient (pH_i_-pH_e_), determined from the experiments represented in Figures 2, 3, are shown by solid gray lines. One example data set is shown in full from period “b” (filled circles) and period “c” (open circles). The Nernstian slope of ideal H^+^ selectivity is shown as a gray dashed line. **(B)** The average slopes calculated from the data in panel (A) are shown as filled (data gathered during period “b”) and open (data gathered during period “c”) circles. The cross represents the average of these data. **(C)** The average x-intercepts calculated from the data in panel (A) are shown as filled (data gathered during period “b”) and open (data gathered during period “c”) circles. The cross represents the average of these data.

In an earlier study we demonstrated that for SLC4A11-expressing oocytes, the relationship between *V*_m_ and the transmembrane pH gradient (64 mV/pH-unit) was close to Nernstian with respect to H^+^ (Myers et al., 2016). Figure 4A shows equivalent data gathered in the presence of 1 mM NH_4_Cl from experiments such as those shown in Figures 2A, 3B. The average slope of the relationship is 71 ± 5 mV/decade (Figure 4B) with an *x*-axis intercept of −1.25 ± 0.04 (Figure 4C). Data from one of the three Figure 3B-style experiments is highlighted in Figure 4A, with black data points taken from period “b” and white data points taken from period “c.” In summary for this section, the slope of the relationship between *V*_m_ and transmembrane pH gradient does not appear to be greatly disturbed by the presence of 1 mM NH_4_Cl, except for the unusual observation that the relationship does not intersect with the origin. The meaning of this observation is explored in Section 3.3.

### 2.4 Comparing the influence of pH_e_ and [NH_3_] on SLC4A11 pK_i_

**FIGURE 5:**
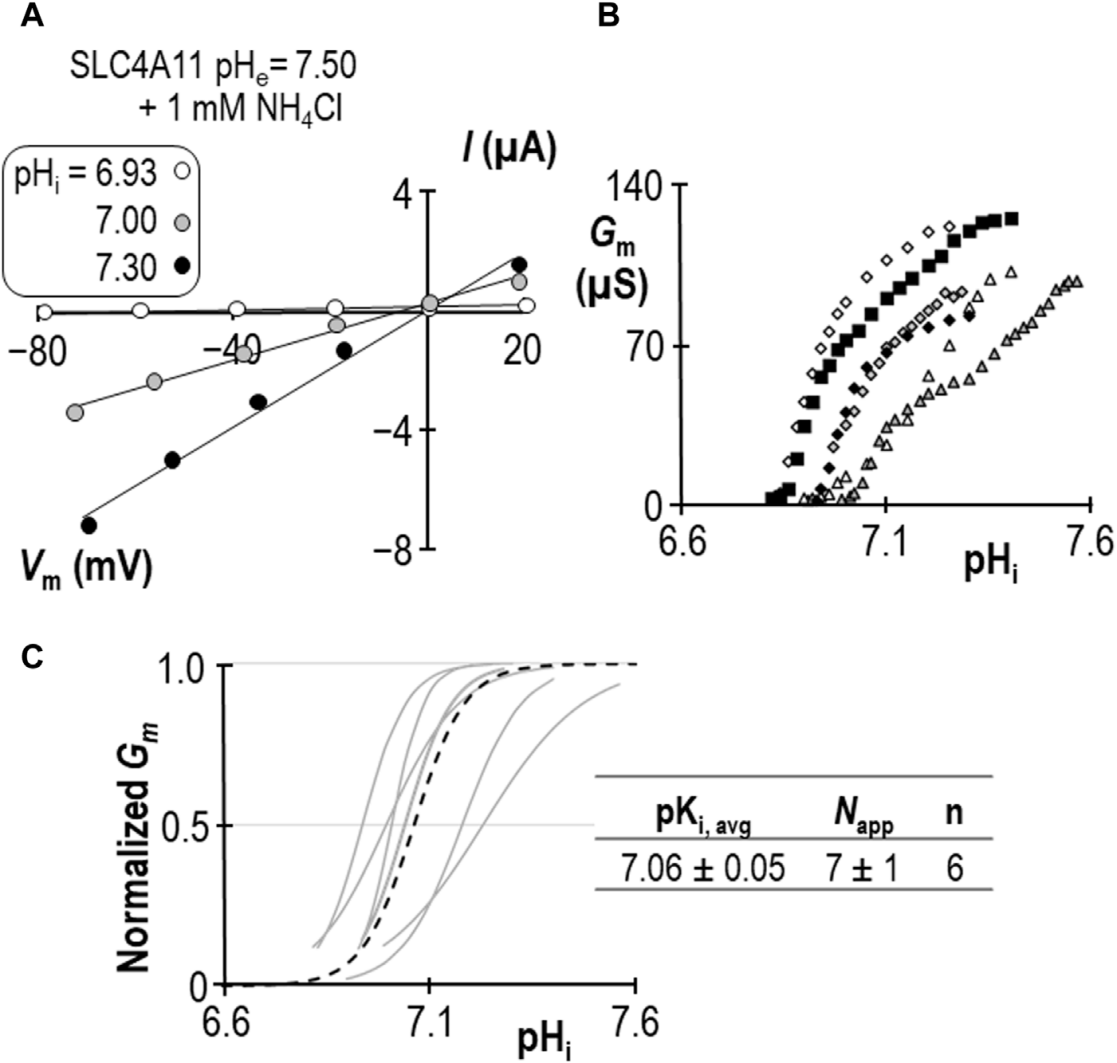
SLC4A11 behavior in the presence of 1 mM NH_4_Cl (extracellular pH = 7.50). **(A)** A representative selection of current-voltage (I-V) relationships gathered from a single SLC4A11-expressing oocyte as intracellular pH (pH_i_) rises. **(B)** The relationship between pH_i_ and slope conductance (*G*_m_) from the full-set of I-V relationships gathered from the SLC4A11-expressing cell shown in panel (A) (black diamonds) and from five other SLC4A11-expressing cells (each represented by its own symbol). **(C)** Best-fit lines for each cell to the Hill equation are shown in gray. The dashed black line represents the Hill equation generated using the average pK_i_ and apparent Hill coefficients (*N*_app_) of the n = 6 replicates, as shown in the inset table.

**FIGURE 6:**
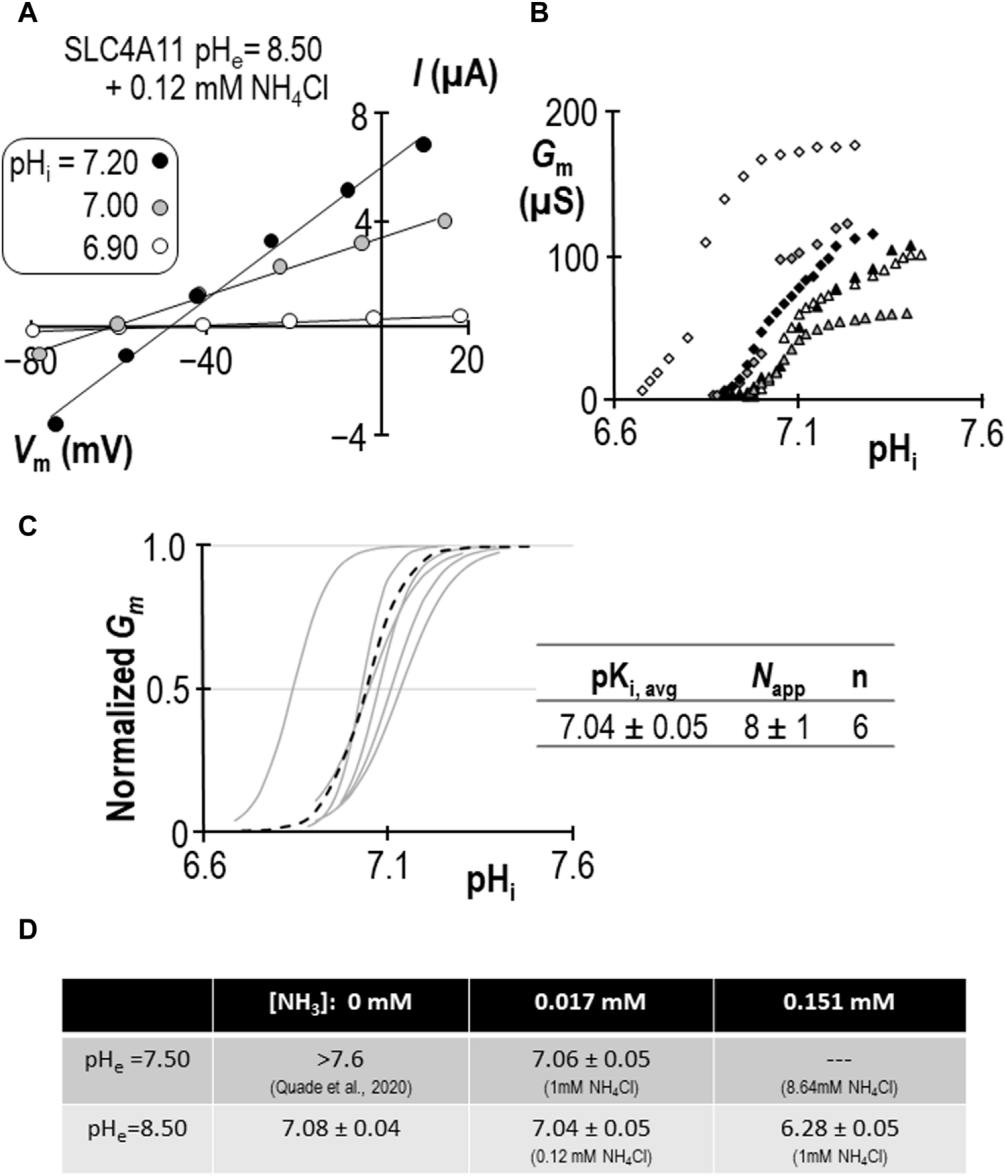
SLC4A11 behavior in the presence of 0.12 mM NH_4_Cl (extracellular pH = 8.50). **(A)** A representative selection of current-voltage (I-V) relationships gathered from a single SLC4A11-expressing oocyte as intracellular pH (pH_i_) rises. **(B)** The relationship between pH_i_ and slope conductance (*G*_m_) from the full-set of I-V relationships gathered from the SLC4A11-expressing cell shown in panel (A) (black diamonds) and from five other SLC4A11-expressing cells (each represented by its own symbol). **(C)** Best-fit lines for each cell to the Hill equation are shown in gray. The dashed black line represents the Hill equation generated using the average pK_i_ and apparent Hill coefficients (*N*_app_) of the n = 6 replicates, as shown in the inset table. **(D)** A summary of the pK_i_ of SLC4A11 detemined under the various conditions tested in this study.

Using a similar work-flow to that described above, we determined the pK_i_ of SLC4A11 at pH_e_ = 7.50 in the presence of 1 mM NH_4_Cl (Figures 5A–C). *G*_m.max_ in this cohort of cells was 104 ± 7 µS, which is not different from the equivalent range reported from cells assayed at pH_e_ = 8.50 + NH_4_Cl (*p* = 0.09: two-tailed, unpaired t-test). Because the ratio of NH_3_:NH_4_^+^ is pH-sensitive, we also determined the pK_i_ of SLC4A11 at pH_e_ = 8.50 in the presence of 0.12 mM NH_4_Cl (Figures 6A–C). In both conditions, although [NH_4_Cl] is different, [NH_3_] is the same (0.017 mM) Figure 6D summarizes the values of pK_i_ that have been determined during this study. We find that pKi at pHe = 7.50 in the presence of 1 mM NH_4_Cl is 7.06 ± 0.05 (Figure 5C), and the pK_i_ at pH_e_ = 8.50 in the presence of 0.12 mM NH_4_Cl is 7.04 ± 0.05 (Figure 6C). These values are not significant different from each other (*p* = 0.68: two-tailed, unpaired t-test).

We can make two additional statistical comparisons: [1] at pH_e_ = 8.50, pK_i_ is not significantly different in the presence or absence of 0.12 mM NH_4_Cl (*P* = 0.50: two-tailed, unpaired t-test), and [2] in the presence of 1 mM NH_4_Cl, raising pH_e_ from 7.50 to 8.50 has a significant acidifying effect on pK_i_ (*p*< 0.01: two-tailed, unpaired t-test). The interpretation of these findings are discussed in Section 3.4.

## 3 Discussion

### 3.1 NH_4_Cl increases SLC4A11 *G*_m_ by shifting pK_i_ in the acidic direction

**FIGURE 7:**
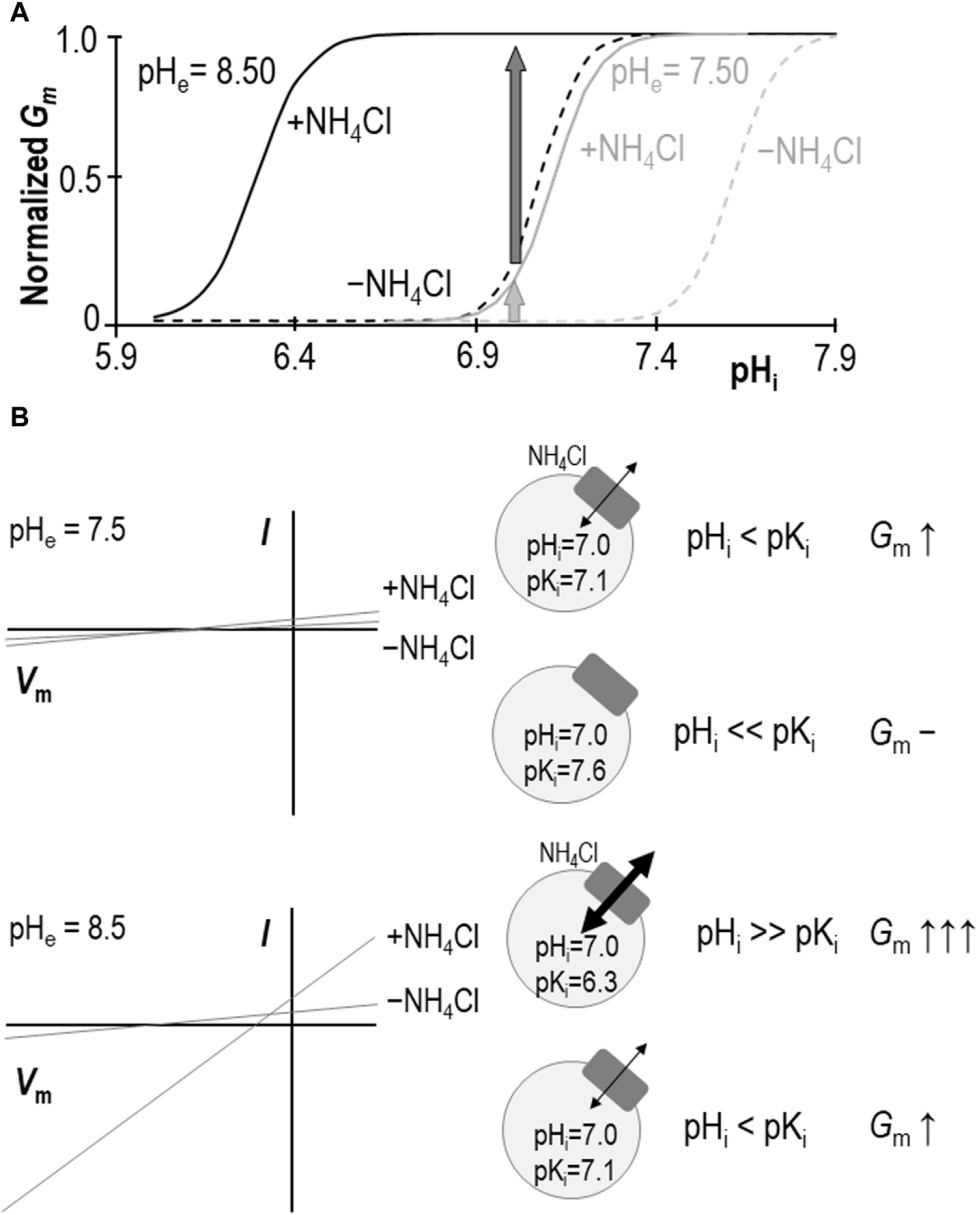
Models of SLC4A11 action in the presence and absence of NH_4_Cl. **(A)** Cartoon representing the pH_i_ versus *G*_m_ relationships for SLC4A11 at pHe 7.50 (gray lines) and 8.50 (black lines) in the presence (solid lines) and absence (dashed lines) of 1 mM NH_4_Cl. **(B)** Cartoon showing how the scheme in panel (A) could explain how NH_4_Cl can achieve greater potency with respect to enhancing SLC4A11-mediated conductance at pH_e_ = 8.5 (lower panel) versus 7.5 (upper panel), as observed in prior studies such as Zhang et al., 2015 or Myers et al., 2016. I-V plots are cartoons, gray circles represent cells expressing SLC4A11 (gray boxes) with black arrows indicating relative magnitudes of conductance.

Our data show that, at pH_e_ = 8.50, the extracellular presence of 1 mM NH_4_Cl results in a significant acidic shift in pK_i_ such that, at almost any value of pH_i_ between 6.0 and 7.3 (i.e., the range bounded by the two ± NH_4_Cl traces: solid and dashed black-lines in Figure 7A), *G*_m_ would be increased. A formal determination of pK_i_ at physiological pH_e_ in the absence of NH_4_Cl has been precluded by a practical limitation on how high we can raise oocyte pH_i_, but we have previously estimated that the value must be more alkaline than 7.6 (Quade et al., 2020). The ability of NH_4_Cl to cause an acidic shift in pK_i_, enables us to determine a pK_i_ range of 7.06 ± 0.05 at pH_e_ = 7.50 in the present study. Thus, we can imagine that, even with the conservative estimate of the NH_4_Cl-free pK_i_, the implication is similar: the presence of NH_4_Cl would cause an increase in *G*_m_ at typical physiological values of pH_i_ (e.g., 7.0–7.3, which is included in the range bounded by the solid and dashed gray-lines in Figure 7A). Critically, this stimulatory effect of NH_4_Cl represents only an increase in *G*_m_ caused by a redefinition of the pH_i_ versus *G*_m_ relationship. Neither *G*_m,max_ nor *N*_app_ are significantly altered by NH_4_Cl (at least as determined at pH_e_ = 8.50) so the redefinition appears to represent a direct acidic-translation of the relationship. Although we are wary of assigning any meaning to the numerical value of *N*_app_ (which is a function of the number of titratable moieties within SLC4A11), the observation of an unchanging *N*_app_ at least implies that mechanism by which SLC4A11 responds to pH_i_ is similarly complex in the absence and presence of NH_4_Cl.

We had once before investigated the role of NH_4_Cl in stimulating SLC4A11 (Myers et al., 2016). We found, as others had before us (Zhang et al., 2015), that the presence of 5 mM NH_4_Cl causes an increase in SLC4A11 *G*_m_ (or *I*_m_ at fixed *V*_m_ in the case of those other studies). However, although we appreciated at that time that SLC4A11 was pH_i_-dependent, we did not consider the possibility that the relationship between pH_i_ and *G*_m_ could be modulated. The addition of 5 mM NH_4_Cl to (even H_2_O-injected) oocytes causes a rapid and robust depolarization (Musa-Aziz et al., 2009). This action in itself is sufficient to drive SLC4A11-mediated H^+^ efflux, resulting in a cellular alkalinization that further activates SLC4A11 (Myers et al., 2016). Thus, in that study, when we increased *G*_m_ to *G*_m.max_ at pH_e_ = 8.50 by adding 5 mM NH_4_Cl and saw no change in that value upon NH_4_Cl removal (maintaining a voltage clamp at 0 mV to mimic the depolarizing effect of NH_4_Cl presence), we assumed that the presence of NH_4_Cl was merely causing *G*_m_ to rise according to the prescribed pH_i_ vs. *G*_m_ relationship. In light of our new data, we reinterpret those data as likely to have been gathered at a pH_i_ value that was greater than the NH_4_Cl-free pK_i_, where *G*_m_ values for both ± NH_4_Cl relationships are close to *G*_m,max_. That is to say that we reinterpret our data in favor of a model in which NH_4_Cl causes a direct rather than indirect allosteric activation of SLC4A11.

### 3.2 Data do not conclusively support a more important role for NH_3_ versus NH_4_^+^ for SLC4A11 action

Models in which SLC4A11 transports NH_3_/NH_4_^+^ favor a NH_3_:*n*H^+^ cotransport mechanism over NH_4_^+^ transport because NH_3_ increases in concentration with rising pH, thereby providing an explanation for the greater stimulation of SLC4A11 currents/conductance at pH_e_ = 8.50 than pH_e_ = 7.50. We might use the same logic to conclude that NH_3_ is more likely than NH_4_^+^ to be the allosterically activating species. However, our data provide an alternative explanation: as shown in Figures 7A,B, at the typical resting pH_i_ range for SLC4A11-expressing oocytes [6.9–7.0: (Quade et al., 2020)], the shift in pK_i_ makes less difference to *G*_m_ at pH_e_ = 7.50 (light gray arrow in Figures 7A,B/upper panel) than at pH_e_ = 8.50 (dark gray arrow in Figures 7A,B/lower panel). In this case it is not necessary to invoke a model in which the abundance of the substrate/activator is pH dependent; NH_4_^+^ (whose fractional abundance is relatively unchanged between pH_e_ 7.50 and 8.50) could be considered equally likely to be the species responsible for the observed stimulation.

Our data do not speak definitively to the nature of the activating species. We believe, because the time course of pK_i_ shift (>10 min: Figure 3A) is much slower than the rate of solution turnover in the bath (<1 min) that the allosteric activation likely requires the activating species to accumulate intracellularly in order to exert its effect on SLC4A11. Because the handling of NH_3_/NH_4_^+^ by oocytes is unusually complex (Musa-Aziz et al., 2009), it is difficult to specifically relate this time course of activation to the accumulation of either species. On the one hand NH_3_ is presumed to be the more membrane permeable of the two species. On the other hand, in contrast to the expected alkalinization observed in mammalian cells exposed to NH_4_Cl, (even H_2_O-injected) oocyte pH_i_ paradoxically acidifies as if NH_4_^+^ is accumulating faster, perhaps entering via non-selective cation channels as NH_3_ is sequestered in sub-membranous granules (Burckhardt and Frömter, 1992). For this reason, it is not clear whether there is a diagnostically useful differential in their rate of accumulation that could point to one species over the other.

### 3.3 SLC4A11 retains H^+^ selectivity in the presence of NH_4_Cl

Another critical parameter that is unchanged in the presence of NH_4_Cl is its H^+^ selectivity, because the *V*_m_ of SLC4A11-expressing cells exhibits a close-to-Nernstian response to changes in the transmembrane pH gradient (Figure 4). This suggests that SLC4A11 remains a selective H^+^ conductor rather than assuming a novel NH_3-_coupling mode. Although previous studies have claimed to provide evidence of NH_3_‐coupled H^+^ transport using a similar approach (Zhang et al., 2015; Kao et al., 2020), we believe that these finding should be interpreted with caution as the approach violates necessary assumptions of reversal potential calculations: chiefly that substrates can only cross the membrane via SLC4A11 (not true for NH_3_) and that *V*_m_ is dominated by the action of SLC4A11 (not true due to the depolarizing action of NH_3_/NH_4_^+^). In the absence of a specific inhibitor for SLC4A11, to distinguish the behavior of the protein from that of the system, the best that such calculations may achieve is a description of the permeability of the system (i.e., SLC4A11 and its membrane environment). One such study concluded that SLC4A11 was capable of both NH_3_:H^+^ cotransport and H^+^ transport (Kao et al., 2020). If so, this is not the typical action of an obligatorily coupled cotransport protein and could equally describe the behavior of a H^+^ conductor in an NH_3_-permeable membrane.

**FIGURE 8:**
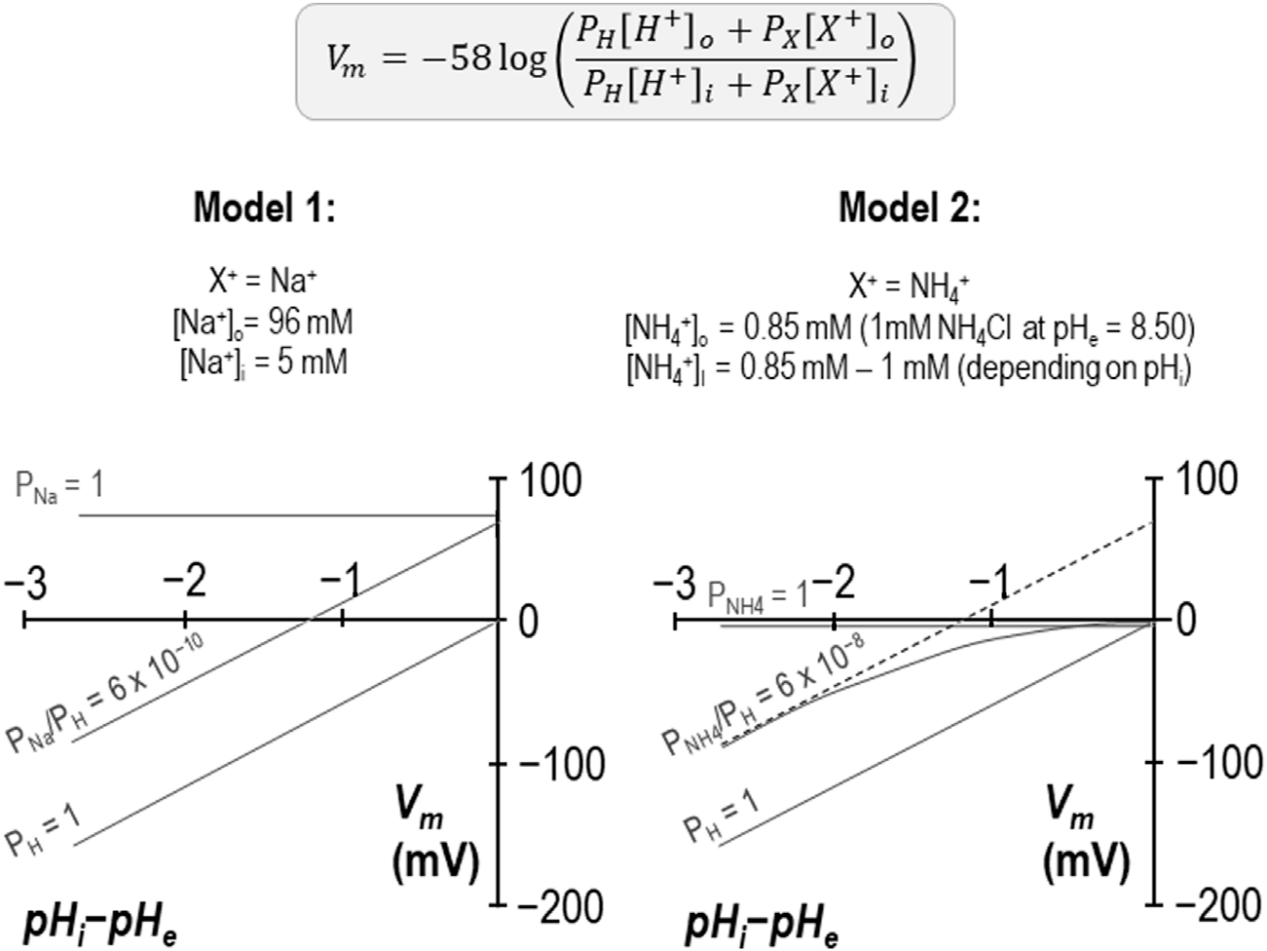
Models of additional, non-H^+^ permeabilities in SLC4A11-expressing cells in the presence of NH_4_Cl Two models based on a modified Goldman-Hodgkin-Katz equation in which the implementation of a small cationic permeability (either Na^+^ in the case of Model 1 or NH_4_^+^ in the case of Model 2) causes an offset in the projected *x*-axis intercept, as observed in our experiments conducted at pH_e_ = 8.50 + 1 mM NH_4_Cl (per Figure 4).

There is one unusual aspect to our reversal potential data that requires further explanation. In previous studies we have found that the relationship between the transmembrane gradient and *V*_m_ for wild-type SLC4A11 crosses the *x*-axis at a value close to zero as expected for a H^+^ conductor. In the present study performed in the presence of NH_4_Cl, we find that the relationship is substantially offset from the origin and intersects the *x*-axis at −1.25 pH-units as if we had underestimated pH_i_ by 1.25 units. This is not impossible, as seven of the ten data sets were gathered while SLC4A11 was mediating H^+^ influx and thus pH immediately below the membrane may have been more acidic than bulk pH_i_ being measured at the tip of our microelectrode, which is impaled deeper into the cell. However, three of these data sets were gathered while SLC4A11 was mediating H^+^ efflux, and exhibited the same offset, so we do not believe that this can be the correct explanation. An alternate explanation is illustrated in Figure 8. Here we show that, if we consider these data as being representative of the system and interpret them using a modified Goldman-Hodgkin-Katz equation, we can reproduce the offset by implementing a small permeability to a depolarizing cation. Two examples are provided: in Model 1, we add a sodium permeability to the system. Because the abundance of H^+^ is so small compared to the abundance of Na^+^, the relative permeability of Na^+^ to H^+^ must be very small not to completely overwhelm *V*_m_. In this instance, P_Na_/P_H_ = 6 × 10^−10^ provides a good fit to our observations. In Model 2 we add an NH_4_^+^ permeability to the system. We assume that [NH_4_Cl] is 1 mM on both sides of the membrane and calculate [NH_4_^+^]_i_ for each value of pH_i_. In this instance P_NH4_/P_H_ = 6 × 10^−8^ provides a good fit for our data. Although the relationship curves off to an asymptote, the initial slope is Nernstian with respect to H^+^, and its projection (dashed gray line) crosses the *x*-axis at −1.25 pH units. We note that the *x*-intercept of our data is also a projection and we do not know whether our data would also curve in a similar way if extended towards the *x*-axis. In any case this is not a critical issue as the relationship can be made to conform to the dashed line if we lower our estimate of [NH_4_Cl]_i_ to 0.1 mM. As either permeability is trivial compared to that of H^+^, it does not appear to be a major confounding factor to our hypothesis. In the absence of a specific blocker, we cannot know whether the additional permeability is intrinsic to SLC4A11 or to the system in general. However, as we have only observed this in the presence of NH_4_^+^, and because NH_4_^+^ is a depolarizing influence in even H_2_O-injected cells, we tentatively suggest that the *x*-axis offset represents the previously described endogenous NH_4_^+^ conductance.

### 3.4 The relationship between pK_i_ shifts caused by extracellular alkalinization versus NH_4_Cl addition

As previously observed, in the absence of NH_4_Cl, a shift of pH_e_ from 7.50 to 8.50, is itself sufficient to acid-shift SLC4A11 pK_i_ by more than 0.5 pH-units (Quade et al., 2020). If we compare SLC4A11 pK_i_ determined at pH_e_ = 7.50 + 1 mM NH_4_Cl to pK_i_ determined at pHe = 8.50 + 0.12 mM NH_4_Cl (conditions in which [NH_3_] is the same and pK_i_ is not different) we may conclude that, in the presence of NH_4_Cl, pK_i_ has become pH_e_-independent and is determined by [NH_3_] alone. In that case we could consider [NH_3_] and pH_e_ as activators that share a common mechanism. If SLC4A11 is less pH_e_ dependent in the presence of NH_4_Cl, that would imply that the stimulatory effect of pH_e_ on SLC4A11 may have limited relevance to its *in vivo* action.

On the other hand, if we compare SLC4A11 pK_i_ determined at pH_e_ = 7.50 + 1 mM NH_4_Cl to pK_i_ determined at pH_e_ = 8.50 + 1 mM NH_4_Cl, values which are ∼0.8 units apart, we may conclude that the phenomenon of pH_e_-dependence is preserved in the presence of 1 mM NH_4_Cl and that the actions of NH_4_Cl and pH_e_ are, at least in part, mechanistically independent and additive. The common ground between these two ways of looking at the data are that the presence of NH_4_Cl alters the relationship between pH_e_ and pK_i_ such that a more acidic pK_i_ can be achieved at a given pH_e_. Interestingly, this is the opposite to a phenomenon that we have observed in relation to certain pathological SLC4A11 mutants such as R125H, in which the mutation causes pK_i_ to become more alkaline at a given pH_e_ (Quade et al., 2022). Unfortunately, the technical limitations on how far we can extend pH_i_ and the low resolution of our pK_i_ assay (due to the large standard error intrinsic to the data sets) currently preclude us from more detailed exploration of the relationship between these activating parameters.

### 3.5 Implications for the physiological role of SLC4A11

The fluid pumping action of corneal endothelial cells requires the action of a basolateral Na/K-ATPase to generate the transmembrane sodium gradient that drives the Na^+^/CO_3_^=^ cotransporter, drawing osmolytes from the stromal fluid to discourage fluid accumulation. The pump is energized by glutaminolysis that feeds α-ketoglutarate into the TCA cycle to generate ATP; a side product of this reaction is NH_3_ (Zhang et al., 2017). We have previously hypothesized that acid-loading by SLC4A11 could be useful to stabilize pH_i_ during robust bicarbonate pump function, responding to Na^+^/CO_3_^=^ cotransporter action by sensing a local rise in pH_i_ (Myers et al., 2016; Nehrke, 2016). Our new data indicate that SLC4A11 is a H^+^ conductor both in the presence and absence of NH_4_Cl and thus that NH_3_/NH_4_^+^ is an allosteric activator rather than a cotransported substrate of SLC4A11. This action is incompatible with a role of corneal endothelial SLC4A11 in mediating the export of excess NH_3_ from glutaminolysis, and incompatible with SLC4A11 being able to harness an outwardly-directed NH_3_ gradient to mediate H^+^ efflux. Because the electrochemical gradient for H^+^ is typically inwardly directed, we predict that a rise in intracellular NH_3_/NH_4_^+^ promotes H^+^ influx independent of any rise in pH_i_. The ability of NH_3_/NH_4_^+^ to acid shift pK_i_ implies that the ability of SLC4A11 to support pump function is potentiated by NH_3_/NH_4_^+^, the generation of which could be considered to be a proxy for the energetic requirements of the pump.

### 3.6 Summary

The presence of NH_4_Cl causes an acidic shift in the pK_i_ of human SLC4A11, which translates to an increase in *G*_m_ at physiological values of pH_i_. The presence of NH_4_Cl does not affect the H^+^ selectivity of SLC4A11, thus we conclude that NH_4_Cl is an allosteric activator of SLC4A11-mediated H^+^ conductance. The influence of increasing [NH_4_Cl] upon SLC4A11 activity is reminiscent of the influence of increasing pH_e_, but further work will be required to determine whether these share a common mechanism.

## 4 Materials and methods

### 4.1 Oocyte preparation and culture

Ovaries were harvested from female *Xenopus laevis* (*Xenopus* Express, Brooksville, FL) in accordance with the protocol approved by the University at Buffalo Institutional Animal Care and Use Committee. Frogs were anesthetized in 0.2% tricaine solution, ovariectomized, and euthanized by exsanguination. Extracted tissue was cut into ∼1 cm^2^ pieces and washed in a Ca^2+^-free solution (82 mM NaCl, 2 mM KCl, 20 mM MgCl_2_, 5 mM HEPES, pH 7.50). Oocytes were liberated by digestion in 2 mg/mL type 1A collagenase solution, and the isolated cells were washed further in the Ca^2+^-free solution to remove the collagenase prior to resuspension in a physiological buffer (ND96: 96 mM NaCl, 2 mM KCl, 1.8 mM CaCl_2_, 1 mM MgCl_2_, 5 mM HEPES, pH 7.50, 200 mOsmol/kg H_2_O). Until experimental use, oocytes were cultured at 18°C in OR3 medium (14 g/L Leiboviz’s L-15 medium powder, 5 mM HEPES, 20 mL/L 100x penicillin-streptomycin, pH 7.50, 200 mOsmol/kg H_2_O).

### 4.2 cRNA preparation and injection

Our starting material was a clone of human SLC4A11-B in pBSXG4 vector. The construct was linearized with HindIII, which cuts at a site downstream of the open reading frame providing a termination point for transcription. The linearized DNA was purified using a MinElute PCR Purification Kit (QIAgen, Germantown, MD) used as a template for cRNA synthesis using the T7 mMESSAGE mMACHINE kit (Invitrogen, Carlsbad, CA). cRNA was purified using an RNeasy MinElute Cleanup KIt (QIAgen). 25 ng of cRNA or H_2_O was injected into each oocyte using a Nanoject programmable injector (Drummond Scientific, Broomall, PA).

### 4.3 Electrophysiology

Oocytes were placed into chamber (RC-3Z: Warner Instruments, Hamden, CT) on an anti-vibration table (Vision IsoStation; Newport Corp., Irvine, CA) and were superfused at 2 mL/min with solutions fed from syringe pumps (Harvard Apparatus, Holliston, MA). Borosilicate glass capillaries (BF200-156-10: Sutter Instrument, Novato, CA) were pulled into microelectrodes (such that they exhibited a tip resistance of 0.1–2 MΩ when filled with saturated KCl solution) using a micropipette puller (P-1000: Sutter Instrument). Oocytes were impaled with two such KCl-filled microelectrodes (one current-passing and one voltage-sensing) connected to an oocyte clamp (OC275: Warner Instruments, Hamden, CT). A bath clamp (725I: Warner Instruments) was used to hold the potential of the chamber fluid at 0 mV. Current-voltage (I-V) plots were gathered in 20 mV, 100 ms steps, returning to the spontaneous membrane potential for 100 ms between each step. H^+^-selective microelectrodes were pulled in the same manner as voltage electrodes but the tips were filled with hydrogen ionophore I/cocktail B (Sigma Aldrich) and backfilled with a solution composed of 40 mM KH_2_PO_4_, 15 mM NaCl, pH 7.0. These electrodes were connected to a dual-channel electrometer (HiZ-223: Warner Instruments). Complete technical details can be found in the 2013 review by Lee, Boron, and Parker (Lee et al., 2013). Signals were digitized via a Digidata 1550 unit and captured using Clampex 10.4 software (Molecular Devices LLC, San Jose) and custom continuous acquisition software (written by Mr. Dale Huffman for Walter Boron’s laboratory at Case Western Reserve University, Cleveland, OH).

### 4.4 Electrophysiology solutions

pH 7.50 solutions contained 96 mM NaCl, 2 mM KCl, 1.8 mM CaCl_2_, 1 mM MgCl_2_, 5 mM HEPES, 200 mOsmol/kg H_2_O. pH 8.50 solutions had the same composition but were buffered with 5 mM Bicine in place of HEPES. NH_4_Cl was added to NH_4_Cl-containing solutions as a powder, and pH was readjusted as necessary.

### 4.5 Data analysis

Data are presented as means ± standard error of the mean. Slope conductance (*G*_m_) was determined from the slope of a linear trendline fit to I-V data in Microsoft Excel. Normalized *G*_m_ data was plotted against pH_i_ (expressed as [OH^−^]) and fit to the Hill equation using the solver add-in of Excel to determine the values of EC_50_ and *N*_app_ that would result in the minimum root square difference between the observed data and the outcome of the Hill equation:

$$\frac{G_{m}}{G_{m.,\max}}=\frac{1}{1+{\left({EC}_{50}/\left[{OH}^{-}\right]\right)}^{N_{app}}}$$

EC_50_ was converted into a value of pK_i_ using the following equation:

$${pK}_{i}=14-\left[-\log\left({EC}_{50}\right)\right]$$

For Figure 3A, we calculated pK_i,app_ by solving the Hill equation for EC_50_ at each pair of *G*_m_ and [OH^−^] (i.e., pH_i_) data points. We assumed that *G*_m,max_ and *N*_app_ were constants: 79 µS and 7 respectively, corresponding to the data gathered from this cell during period “b.” Calculations of [NH_3_] at a given pH value and [NH_4_Cl] assume a pK_a_ for the NH_3_/NH_4_^+^ equilibrium of 9.25.

Statistical analysis was performed in Excel using unpaired t-tests, one- or two-tailed as necessary. For multiple comparison, ANOVA was performed using MiniTab software.

## Data Availability

The datasets presented in this study can be found in online repositories. The names of the repository/repositories and accession number(s) can be found below: https://doi.org/10.6084/m9.figshare.25928233.v1.
